# *Caenorhabditis* Intervention Testing Program: the farnesoid X receptor agonist obeticholic acid does not robustly extend lifespan in nematodes

**DOI:** 10.17912/micropub.biology.000257

**Published:** 2020-05-27

**Authors:** Mackenzie L Morshead, Christine A Sedore, E Grace Jones, David Hall, W Todd Plummer, Theo Garrett, Mark Lucanic, Max Guo, Monica Driscoll, Patrick C Phillips, Gordon Lithgow

**Affiliations:** 1 The Buck Institute for Research on Aging, Novato, California 94945, USA; 2 Institute of Ecology and Evolution, University of Oregon, Eugene, Oregon 97403, USA; 3 Division of Aging Biology, National Institute on Aging, Bethesda, Maryland 20892, USA; 4 Department of Molecular Biology and Biochemistry, Rutgers University, Piscataway, New Jersey 08854, USA

**Figure 1:**
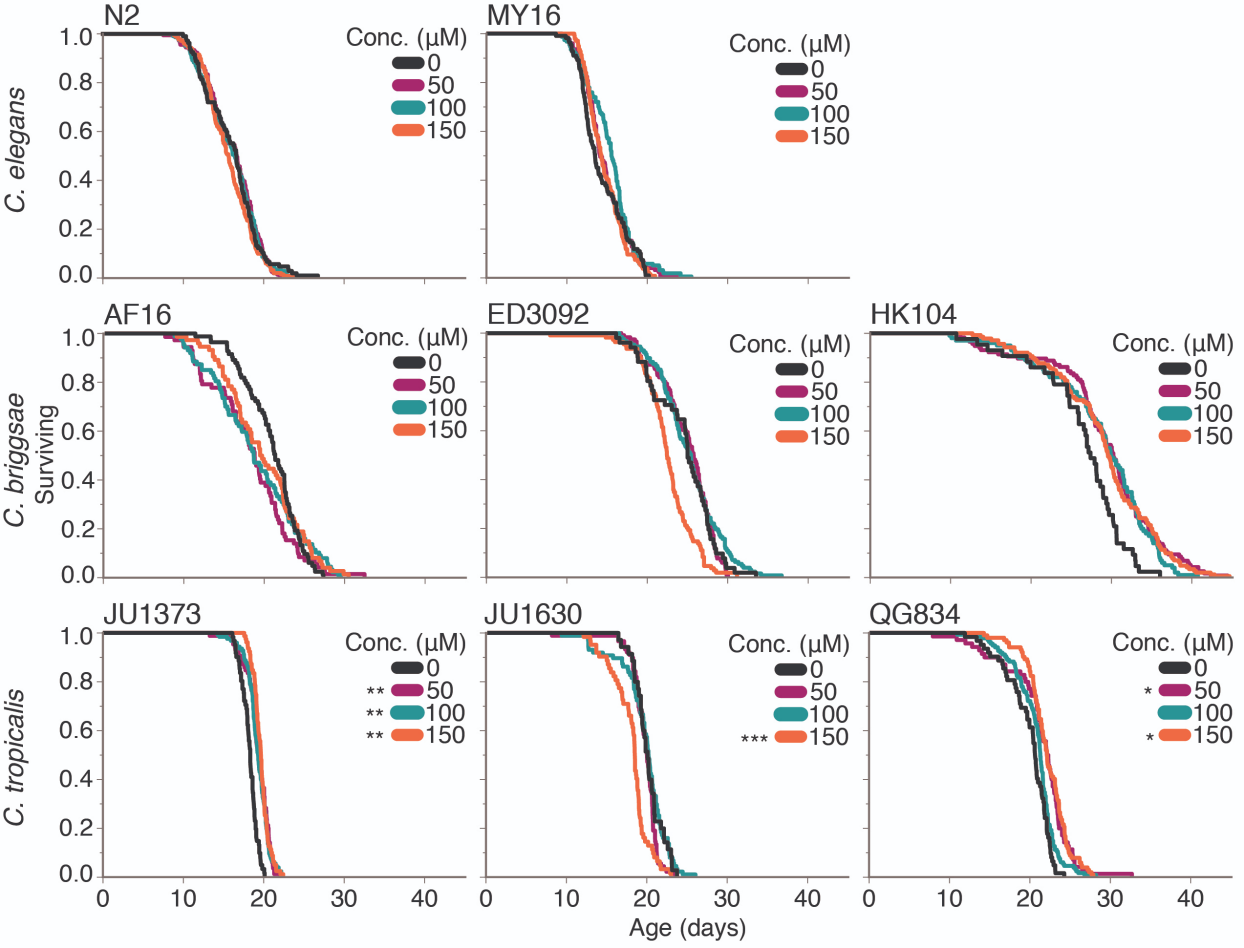
**Survival under adult obeticholic acid exposure:** Survival curves for *C. elegans* strains N2 and MY16, *C. briggsae* strains AF16, ED3092 and HK104, and *C. tropicalis* strains JU1373, JU1630 and QG834 exposed to obeticholic acid at various concentrations starting on day one of adulthood. Each line represents lifespan data from multiple replicates. JU1373 lifespan differed significantly from control (mean=18.2 days) at 50 µM, 100 µM, and 150 µM (mean=19.2, 19.2 and 19.6, days; *p*=0.006, 0.008 and 0.001, respectively). JU1630 survival differed significantly from control (mean=20.2 days) at 150 µM (mean=18.1 days; *p*<0.001). QG834 differed significantly from control (mean=19.9 days) at 50 µM and 150 µM (mean=21.3 and 22.2 days; *p*=0.01 and 0.04, respectively). Statistical comparisons are from the Cox proportional hazards (CPH) model with mixed effects using the coxme package v2.2-10 in R (Therneau 2018; R Core Team 2019). Asterisks represent *p*-values from the CPH model such that ***p<0.001, **p<0.01, and *p<0.05.

## Description

The *Caenorhabditis* Intervention Testing Program (CITP) is a multi-institutional, National Institute on Aging (NIA)-funded consortium. The goal of the program is to identify chemical compounds that extend lifespan robustly and reproducibly across genetically diverse *Caenorhabditis* strains (Lucanic *et al.* 2017). The CITP test compounds are selected if they are consistently highly ranked via computational prediction for lifespan or healthspan effects (Coleman-Hulbert *et al.* 2019), if they are predicted or known to interact with known lifespan-regulating pathways, or if they have previously been reported as extending lifespan or healthspan in laboratory animals. Obeticholic acid is an analog of the natural bile acid chenode oxycholic acid, which acts as an agonist of the farnesoid X receptor (FXR) (Neuschwander-Teri *et al.* 2015), a nuclear receptor (NR) closely involved with hepatic triglyceride homeostasis. Obeticholic acid is most commonly used to treat the autoimmune liver disease, primary biliary cholangitis. The most likely homolog of FXR in *C. elegans* is DAF-12, which can bind and be activated by human bile acids (Held *et al.* 2006; Zhi *et al.* 2011). DAF-12 modulation is of particular interest because it is closely linked to dauer formation, lifespan extension, and metabolism homeostasis (Antebi 2015).

We assayed lifespan in response to different concentrations of obeticholic acid exposure in three *Caenorhabditis* species using the flatbed scanner-based Automated Lifespan Machine (ALM) workflow previously published (Banse *et al.* 2019). To summarize, the worms were age synchronized by egg-lays on standard 60 mm diameter Nematode Growth Media (NGM) plates with lawns of *Escherichia coli* OP50-1, and transferred to compound-treated 38 mm NGM plates containing 51 μM 5-Fluoro-2′-deoxyuridine (FUdR) at a density of 50 worms per plate on day one of adulthood. For treatment plates, we used standardized protocols (*Caenorhabditis* Intervention Testing Program 2020); in short, obeticholic acid (Apexbio Technology) was dissolved in dimethyl sulfoxide (DMSO) and diluted appropriately such that addition of 7.5 µl of stock solution and 125 µl water for 35 mm diameter plates, and 17.5 µl of stock solution and 232.5 µl water for 50 mm diameter scanner plates would generate 50, 100, and 150 µM final obeticholic acid concentrations. For control plates, DMSO was added instead of stock solution using the same method. The worms were maintained at 20ºC and transferred to new treatment plates again on day two of adulthood. One week after age-synchronization (day five of adulthood for *C. elegans* and *C. briggsae*, day four for *C. tropicalis*), the worms were transferred to compound-treated scanner plates and loaded onto the ALM. At this point, automated survival monitoring began, and the scanner data was collected and analyzed using Lifespan Machine software *(*https://github.com/nstroustrup/lifespan; Stroustrup *et al.*. 2013).

Our results indicate that obeticholic acid does not have a consistent beneficial effect on lifespan in any of the *C. elegans* or *C. briggsae* strains tested at the concentrations used. Although we did see some significant differences from the control for some of the concentrations in the *C. tropicalis* strains, overall the difference was not robust. We actually saw a significant decrease in lifespan in *C. tropicalis* JU1630, a weakly significant increase in *C. tropicalis* QG834 at some concentrations, and a relatively significant increase in *C. tropicalis* JU1373, but with only a 5.7-7.9% change in mean survival from the control (Fig. 1). In summary, our results do not indicate a robust effect of obeticholic acid on *Caenorhabditis* lifespan. This conclusion is based upon two biological replicates at each concentration performed in one lab, resulting in an average of 104 individuals measured per strain and concentration, and should be considered preliminary. The effect on lifespan in this study may pertain to a lack of physiological relevance of obeticholic acid to *Caenorhabditis.* Obeticholic acid was of interest to the CITP because of its effect on the mammalian NR FXR. Although DAF-12 has been identified as a potential *Caenorhabditis* homolog of FXR and other bile acids have been shown to bind with DAF-12 (Zhi *et al.* 2011), it is possible that obeticholic acid was not able to bind with high affinity to the receptor, therefore eliciting little to no effect on lifespan. Alternatively, obeticholic acid may be rapidly metabolized in *Caenorhabditis*.
